# A Disease of the Past in the Present: From Trench Foot to Critical Care With Severe Non-freezing Cold Injury

**DOI:** 10.7759/cureus.96139

**Published:** 2025-11-05

**Authors:** Nuno Prucha Leite, David Costa, Marta Rebocho Alves, João Abranches Carvalho, Luís Teles

**Affiliations:** 1 Department of Intensive Care Medicine, Local Health Unit of Entre Douro e Vouga, Santa Maria da Feira, PRT

**Keywords:** aeromonas hydrophila, critical care, gram-negative bacteremia, hypothermia, non-freezing cold injury, sepsis, trench foot

## Abstract

Non-freezing cold injury (NFCI), historically described as trench foot, is a rare but potentially life-threatening condition resulting from prolonged exposure to cold, wet environments at near-freezing temperatures. We present the case of a previously healthy 21-year-old man found partially submerged for three days, who developed profound hypothermia, shock, altered consciousness, and extensive soft tissue compromise. Laboratory evaluation revealed multiorgan dysfunction, and brain computed tomography (CT) demonstrated diffuse cerebral edema. Blood cultures became positive within seven hours, isolating *Aeromonas hydrophila* and *Pseudomonas* species, confirming fulminant Gram-negative bacteremia secondary to soft tissue infection. After a period of intensive supportive care, the patient improved clinically and was transferred to a burn unit for surgical management. This case highlights that NFCI is not confined to historical military contexts but remains a relevant contemporary clinical challenge. Early recognition, aggressive supportive care, and timely surgical intervention are crucial to improving survival, limiting sepsis progression, reducing tissue loss, and preventing long-term disability.

## Introduction

Non-freezing cold injury (NFCI), also known as trench foot or immersion foot, predominantly affects the distal extremities, but prolonged exposure to cold and wet conditions at temperatures marginally above freezing (0-15°C) may lead to more diffuse tissue involvement [1]. First described in the early 19th century and extensively reported during World War I, NFCI results in damage to soft tissues, peripheral nerves, and the microvasculature. Its pathophysiology is incompletely understood but appears to involve impaired circulatory control and direct microvascular injury, with immobility and malnutrition acting as contributory factors [2-4].

In contrast to frostbite, which requires subzero temperatures, NFCI develops at higher temperatures and is primarily driven by neurovascular and microcirculatory dysfunction [5,6]. Clinical manifestations range from mild sensory disturbances to severe tissue compromise, often complicated by secondary infection or systemic illness. Although most cases are subacute, rare presentations with multiorgan dysfunction and critical illness have been described [2,3,7].

Historically linked to military conflicts, NFCI continues to occur in civilian populations exposed to prolonged cold and wet environments, such as homeless individuals, outdoor workers, and participants in large gatherings like music festivals [2,3]. Management remains largely supportive, with emphasis on rewarming, analgesia, wound care, and the prevention of infection and long-term disability [4].

Here, we describe a rare case of severe NFCI in a previously healthy young adult who developed profound hypothermia, multiorgan dysfunction, and bloodstream infection with *Aeromonas hydrophila* and *Pseudomonas *species, requiring intensive care and subsequent surgical management [1,3,8]. This case highlights that NFCI is not confined to historical accounts but can present acutely with life-threatening complications, demanding prompt recognition and coordinated multidisciplinary care.

## Case presentation

A 21-year-old previously healthy man was reported missing for three days and was found partially submerged in swamp water containing a mix of fresh, salt, and stagnant water, with only his upper chest and head above the surface. The pre-hospital emergency team documented profound hypothermia, coma (Glasgow Coma Scale score 5: E1V1M3), and respiratory failure, prompting immediate intubation. Invasive rewarming was initiated, intravenous fluids were administered during transport, and muddy gastric contents were aspirated via a nasogastric tube.

**Figure 1:**
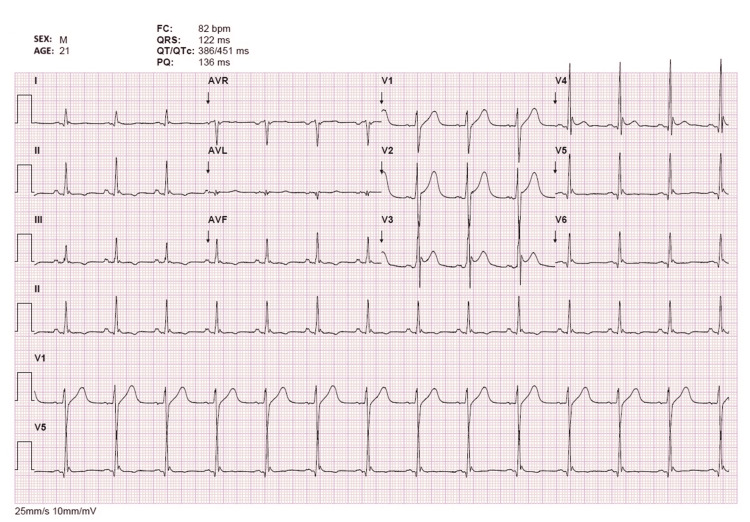
Electrocardiogram at emergency department admission Twelve-lead electrocardiogram demonstrating sinus rhythm with prominent Osborn (J) waves visible across multiple leads, a hallmark finding of severe hypothermia. T-wave inversion is also observed in the inferior leads (II, III, aVF). No significant arrhythmias were recorded at the time.

At emergency department admission, he presented hypotensive (89/56 mmHg), with bilaterally miotic but reactive pupils, multiple abrasions at different stages of healing (sparing the pubic area), cellulitis of the lower limbs with signs of prolonged saltwater immersion, and extensive skin maceration. Laboratory studies revealed severe metabolic acidosis with acidemia in the absence of hyperlactatemia, acute kidney injury (Kidney Disease: Improving Global Outcomes (KDIGO) stage 2) with multiple electrolyte disturbances, marked rhabdomyolysis, and elevated inflammatory markers, accompanied by leukopenia and neutropenia (Table 1). Toxicology screening was positive for amphetamines and tetrahydrocannabinol. Electrocardiography demonstrated Osborn waves (Figure 1), and brain computed tomography (CT) revealed diffuse cerebral edema consistent with hypoxic-ischemic encephalopathy.

**Table 1:** Initial laboratory and microbiological findings at emergency department admission The table summarizes key laboratory and microbiological results at admission, including arterial blood gases, complete blood count, renal function, electrolytes, muscle injury markers, inflammatory parameters, and microbiology. Asterisks indicate values outside the normal reference range. Reference intervals may vary slightly depending on institutional laboratory standards.

Parameter	Result	Unit	Reference range
pH	7.02*	-	7.35-7.45
Bicarbonate (HCO₃⁻)	12.7*	mmol/L	22-28
Lactate	1.3	mmol/L	<2.0
Hemoglobin	13.2*	g/dL	13.5-17.5
White blood cells	1.3*	×10⁹/L	4.0-11.0
Platelets	112*	×10⁹/L	150-450
Creatinine	1.9*	mg/dL	0.6-1.1
Urea	248*	mg/dL	15-40
Sodium	121*	mmol/L	136-145
Potassium	3.4*	mmol/L	3.5-5.1
Chloride	86*	mmol/L	98-107
Phosphate	7.9*	mg/dL	2.3-4.7
Magnesium	1.3*	mmol/L	0.66-1.07
Creatine kinase	9357*	U/L	30-200
Myoglobin	10194*	ng/mL	<140
Glucose	105	mg/dL	70-105
C-reactive protein	222*	mg/L	<5.0
Procalcitonin	13.6*	ng/mL	<0.5
Blood cultures (first and second sets, aerobic; first set, anaerobic)	*Aeromonas hydrophila* and *Pseudomonas *species (positive at seven hours). Resistance profile: *Aeromonas hydrophila* susceptible to all tested antibiotics (standard dose). *Pseudomonas *species susceptible to increased exposure to ceftazidime, piperacillin/tazobactam, and levofloxacin and susceptible to amikacin and tobramycin	-	Negative

**Figure 2:**
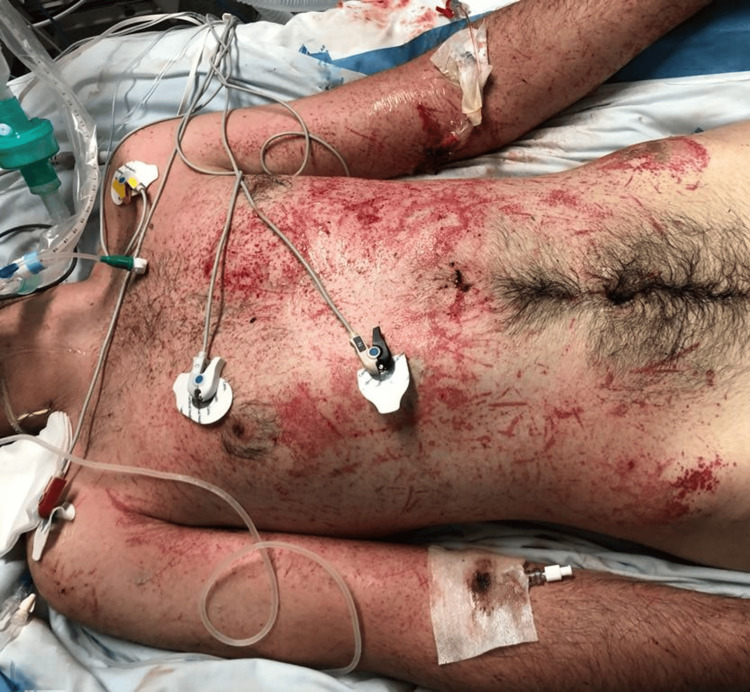
Anterior torso showing the cutaneous manifestations of severe non-freezing cold injury Clinical photograph obtained in the early days after intensive care unit admission. The image demonstrates diffuse erythema with multiple abrasions and macerated skin lesions involving the anterior torso.

**Figure 3:**
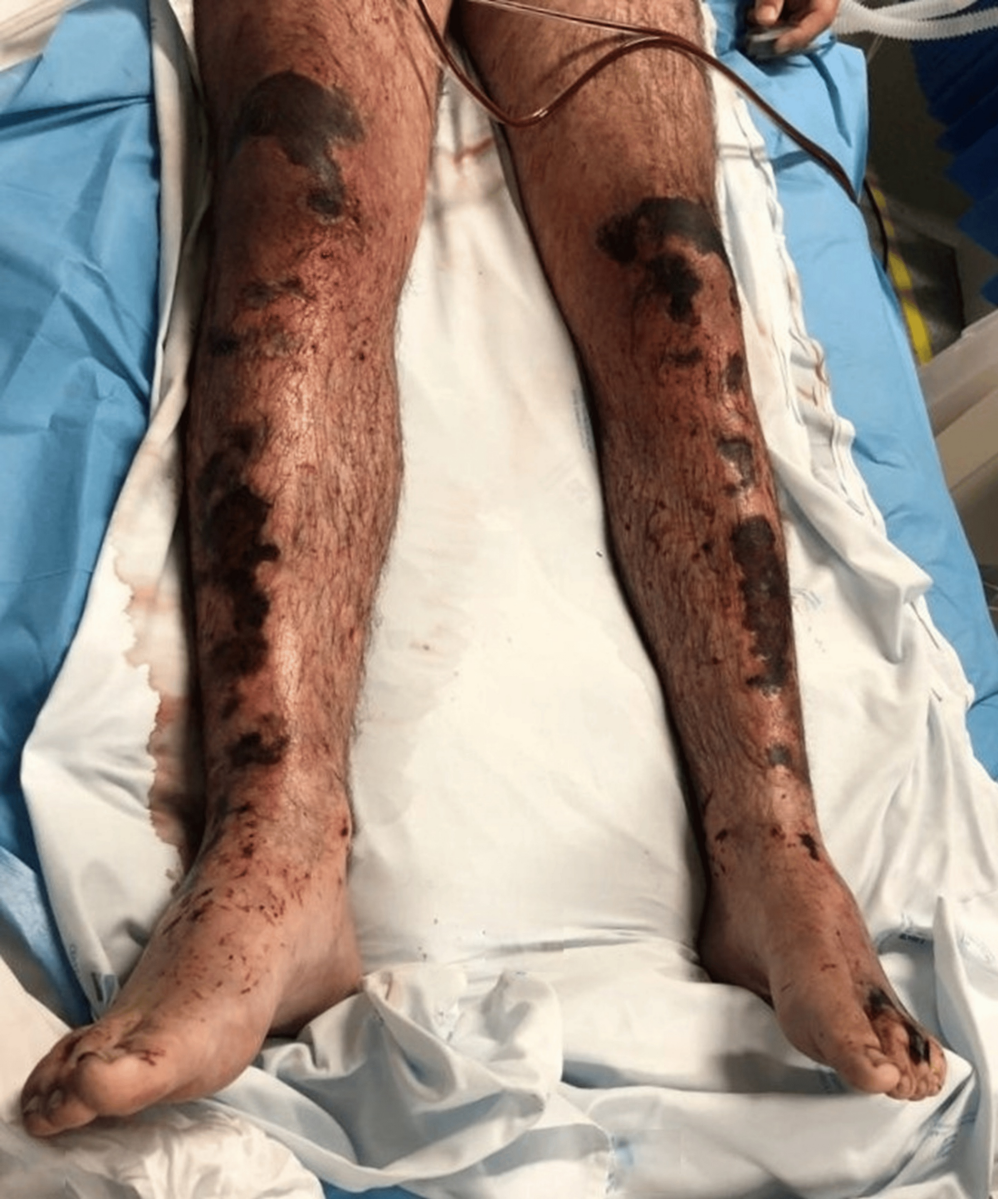
Lower limbs demonstrating extensive ischemic and necrotic changes due to severe non-freezing cold injury Clinical photograph showing diffuse cellulitis with areas of soft tissue necrosis and ischemic discoloration, predominantly involving the distal segments of both lower limbs, consistent with severe non-freezing cold injury. The photograph was taken in the early days after intensive care unit admission.

He was admitted to the intensive care unit (ICU), where treatment included invasive mechanical ventilation, continuous renal replacement therapy, vasopressor support, and a substantially positive fluid balance due to increased evaporative and exudative losses across disrupted skin barriers, consistent with multiorgan failure requiring full supportive care. The affected body surface area was estimated at 70% by plastic surgery assessment. Broad-spectrum empiric antibiotics (doxycycline, levofloxacin, meropenem) were initiated at admission. Blood cultures became positive within seven hours, isolating *Aeromonas hydrophila *and *Pseudomonas *species, confirming fulminant Gram-negative bacteremia. Surgical debridement was initially deferred due to hemodynamic instability and coagulopathy. During the ICU stay, cerebral edema improved on serial imaging, and clinical stabilization allowed transfer after six days to a specialized burn unit for the management of extensive cutaneous lesions (Figures 2-3).

During an 84-day hospitalization in the burn unit, he underwent 34 sessions of hydrotherapy for wound care and five major surgical procedures, including extensive eschar excision, muscle debridement, transmetatarsal amputation of four toes, a free latissimus dorsi myocutaneous flap for tibial coverage, and multiple split-thickness skin grafts. Serial cultures subsequently isolated multidrug-resistant *Aeromonas caviae*, *Stenotrophomonas maltophilia*, *Pseudomonas aeruginosa*, and *Providencia stuartii*, and antimicrobial susceptibility testing guided escalation to targeted therapy with ceftazidime-avibactam and colistin.

He was discharged after nearly three months of hospitalization in the burn unit. At the six-month follow-up, he demonstrated full independence in activities of daily living (Barthel Index=100/100), remained without motor deficits, and continued lower limb rehabilitation and psychological support.

## Discussion

Although NFCI was historically associated with military campaigns, recent reports confirm its persistence in contemporary civilian contexts. Documented cases increasingly involve vulnerable populations such as homeless individuals, outdoor laborers, and participants in prolonged outdoor events exposed to cold and wet conditions without adequate protection [2,3]. The pathophysiological mechanisms of NFCI remain only partially understood but are thought to involve sustained vasoconstriction, endothelial injury, and microcirculatory impairment, with immobility and malnutrition serving as key aggravating factors [3,4]. Prolonged cold water exposure can trigger systemic effects through sustained peripheral vasoconstriction, hypoxia, and reperfusion injury, leading to myocyte necrosis and rhabdomyolysis. Endothelial dysfunction and capillary leak contribute to tissue edema and may underlie cerebral edema observed in severe hypothermic states [1,3,6].

Most contemporary reports describe NFCI as a subacute condition characterized by neuropathic pain, paresthesia, and skin maceration, with progression to necrosis or systemic illness being uncommon [2,3,6]. The present case illustrates an extreme and rarely documented presentation in which NFCI was accompanied by profound hypothermia, shock, acute kidney injury, hypoxic-ischemic encephalopathy, and bacteremia, requiring intensive care. This highlights that, under conditions of prolonged exposure and systemic compromise, NFCI can evolve beyond localized tissue injury to life-threatening multiorgan dysfunction.

Superimposed infection is an underrecognized but clinically significant complication of NFCI. In this case, blood cultures became positive within seven hours, reflecting the fulminant nature of Gram-negative bacteremia arising from compromised soft tissues. The isolation of *Aeromonas hydrophila*, a freshwater-associated pathogen commonly linked to traumatic wound infections, together with *Pseudomonas *species, is noteworthy, as microbiological characterization is seldom detailed in NFCI reports [2,8]. Both *Aeromonas *and *Pseudomonas *species thrive in stagnant or brackish water and can invade through macerated or hypoxic tissue, especially following prolonged immersion and microvascular injury. Their presence in this case likely reflects environmental contamination of compromised skin barriers, leading to early systemic dissemination. The subsequent emergence of multidrug-resistant organisms (*Aeromonas caviae*, *Stenotrophomonas maltophilia*, *Pseudomonas aeruginosa*, *Providencia stuartii*) further illustrates the infectious complexity of this condition, prompting escalation to targeted antimicrobial therapy.

Management of NFCI is largely supportive, emphasizing prevention, gradual passive rewarming, analgesia, and meticulous wound care, while rapid warm water immersion should be avoided. Current recommendations derive primarily from expert consensus, with no universally standardized protocols and considerable variability in practice [4,6]. In our patient, survival required early broad-spectrum antibiotics, advanced organ support, and staged surgical intervention, including amputations, flap reconstruction, and grafting. The need for such complex procedures underscores the potential morbidity of severe NFCI, even with optimal care.

Long-term sequelae, including chronic neuropathic pain, cold sensitivity, and complex regional pain syndrome, are well documented in NFCI survivors [1,3,6]. At six months, our patient remained autonomous and cognitively intact but required ongoing rehabilitation for persistent lower limb sequelae and continued psychological support, underscoring the lasting burden of disease beyond acute survival.

This case highlights that NFCI is not merely a historical condition but a rare yet clinically relevant entity capable of presenting with fulminant critical illness. Its clinical spectrum is broader than often appreciated, extending from mild neuropathic pain to septic shock and multiorgan failure. Clinicians should maintain a high index of suspicion in patients with prolonged cold water exposure, particularly within vulnerable populations, as timely recognition, aggressive multidisciplinary management, and attention to infectious risk are essential to improving survival and reducing long-term disability.

## Conclusions

This case illustrates a rare but fulminant presentation of NFCI, progressing from prolonged cold water immersion to septic shock and multiorgan dysfunction. It reinforces that NFCI is not confined to historical wartime accounts but remains a contemporary condition affecting vulnerable civilian populations. Despite advances in supportive care, severe NFCI carries substantial morbidity. Prompt recognition, coordinated multidisciplinary critical care, and timely surgical intervention are essential to improving survival, limiting tissue loss, and reducing long-term disability. Increased awareness and preventive education among at-risk civilian groups may help reduce the incidence and severity of such injuries.
